# Supplementary material for: Identification of photoperiod-induced specific miRNAs in the adrenal glands of Sunite sheep (Ovis aries)
Source: Front Vet Sci. 2022 Jul 22;9:888207. doi: 10.3389/fvets.2022.888207 (PMC9354845; doi:10.3389/fvets.2022.888207)
Supplement: Supplementary file 1 [file Data_Sheet_1.ZIP › Supplementary file/miRDeep2 file.zip!/miRDeep2 file/Know_Structure/LP42A1/oar-mir-299.pdf]

miRBase precursor : oar-mir-299
Total read count : 210
oar-miR-299-5p read count : 208
oar-miR-299-3p read count : 2
remaining reads : 0

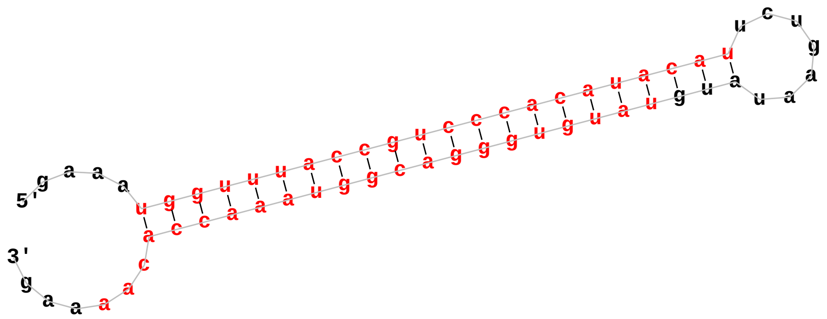

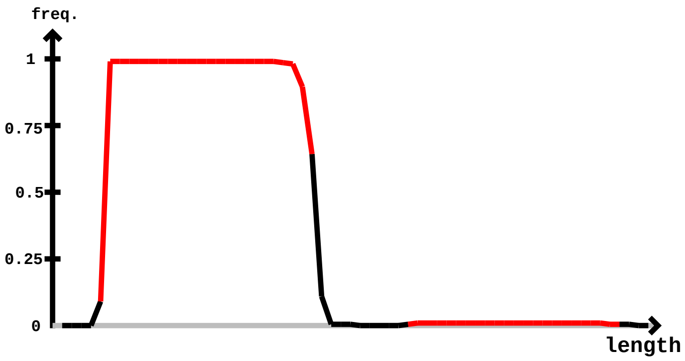

oar-miR-299-5p

oar-miR-299-3p

```
5'- gaaaugguuuaccgucccacauacauucugaauauguaugugggacgguaaaccacaaaag  -3'    exp
    ....((((((((((((((((((((((.......))))))))))))))))))))))......    reads   mm   sample
    ...augguuuaccgucccacauac.....................................    4       0    seq
    ...augguuuaccgucccacauaca....................................    15      0    seq
    ....ugguuuaccgucccacau.......................................    1       0    seq
    ....ugguuuaccgucccacaua......................................    1       0    seq
    ....ugguuuaccgucccacauac.....................................    14      0    seq
    ....ugguuuaccgucccacauaca....................................    38      0    seq
    ....ugguuuaccgucccacauacau...................................    112     0    seq
    ....ugguuuaccgucccacauacauu..................................    15      0    seq
    ....ugguuuaccgucccacauacauA..................................    7       1    seq
    ....ugguuuaccgucccacauacauucug...............................    1       0    seq
    ...................................guaugugggacgguaaaccacaaa..    1       0    seq
    ....................................uaugugggacgguaaaccac.....    1       0    seq
```
